# New Insights About the Sticking Region in Back Squats: An Analysis of Kinematics, Kinetics, and Myoelectric Activity

**DOI:** 10.3389/fspor.2021.691459

**Published:** 2021-06-08

**Authors:** Stian Larsen, Eirik Kristiansen, Roland van den Tillaar

**Affiliations:** Department of Sports Sciences and Physical Education, Nord University, Levanger, Norway

**Keywords:** strength, electromyography, sticking point, inverse dynamics analysis, powerlifting

## Abstract

The aim of this study was to investigate barbell, joint kinematics, joint kinetics of hip, knee, and ankle in tandem with myoelectric activity around the sticking region in three-repetition maximum (3-RM) back squats among recreationally trained lifters. Unlike previous literature, this study also investigated the event of first-peak deacceleration, which was expected to be the event with the lowest force output. Twenty-five recreationally trained lifters (body mass: 70.8 ± 10.5, age: 24.6 ± 3.4, height: 172 ± 8.5) were tested in 3-RM back squats. A repeated one-way analysis of variance showed that ground reaction force output decreased at first peak deacceleration compared with the other events. Moreover, torso forward lean, hip moment arm, and hip contribution to total moment increased, whereas the knee moment arms and moment contribution to total moment decreased in the sticking region. Also, stable moment arms and moment contributions to total moment were observed for the ankle in the sticking region. Furthermore, the knee extensors together with the soleus muscle decreased myoelectric activity in the post-sticking region, while the gluteus maximus and biceps femoris increased myoelectric activity in the post-sticking region. Our findings suggest that the large hip moment arms and hip contributions to total moment together with a lower myoelectric activity for the hip extensors contribute to a poor biomechanical region for force output and, thereby, to the sticking region among recreationally trained lifters in 3-RM back squats.

## Introduction

When it comes to resistance training, squatting is one of the most commonly and widely used exercises for the lower body, since it involves full range of motion and strength of the knee, hip, and ankle joint (van den Tillaar and Larsen, 2020). The squat is commonly used in strength and conditioning preparation in a variety of sports, including the competitive sports of powerlifting and weightlifting, as well as rehabilitation. There are many variations of squats (Glassbrook et al., 2017), and many studies have investigated different variations of squat technique to enhance squatting performance (Benz and West Chester, 1989; Fry et al., 1993; Wretenberg et al., 1996; Anderson et al., 1998; Mccaw and Melose, 1999; Escamilla et al., 2001a,b; Pereira et al., 2010; Bryanton et al., 2012; Swinton et al., 2012; Saeterbakken et al., 2016; Glassbrook et al., 2019; Lahti et al., 2019; van den Tillaar, 2019; Maddox et al., 2020; van den Tillaar and Larsen, 2020; van den Tillaar et al., 2020; Maddox and Bennett, 2021).

**Figure 1:**
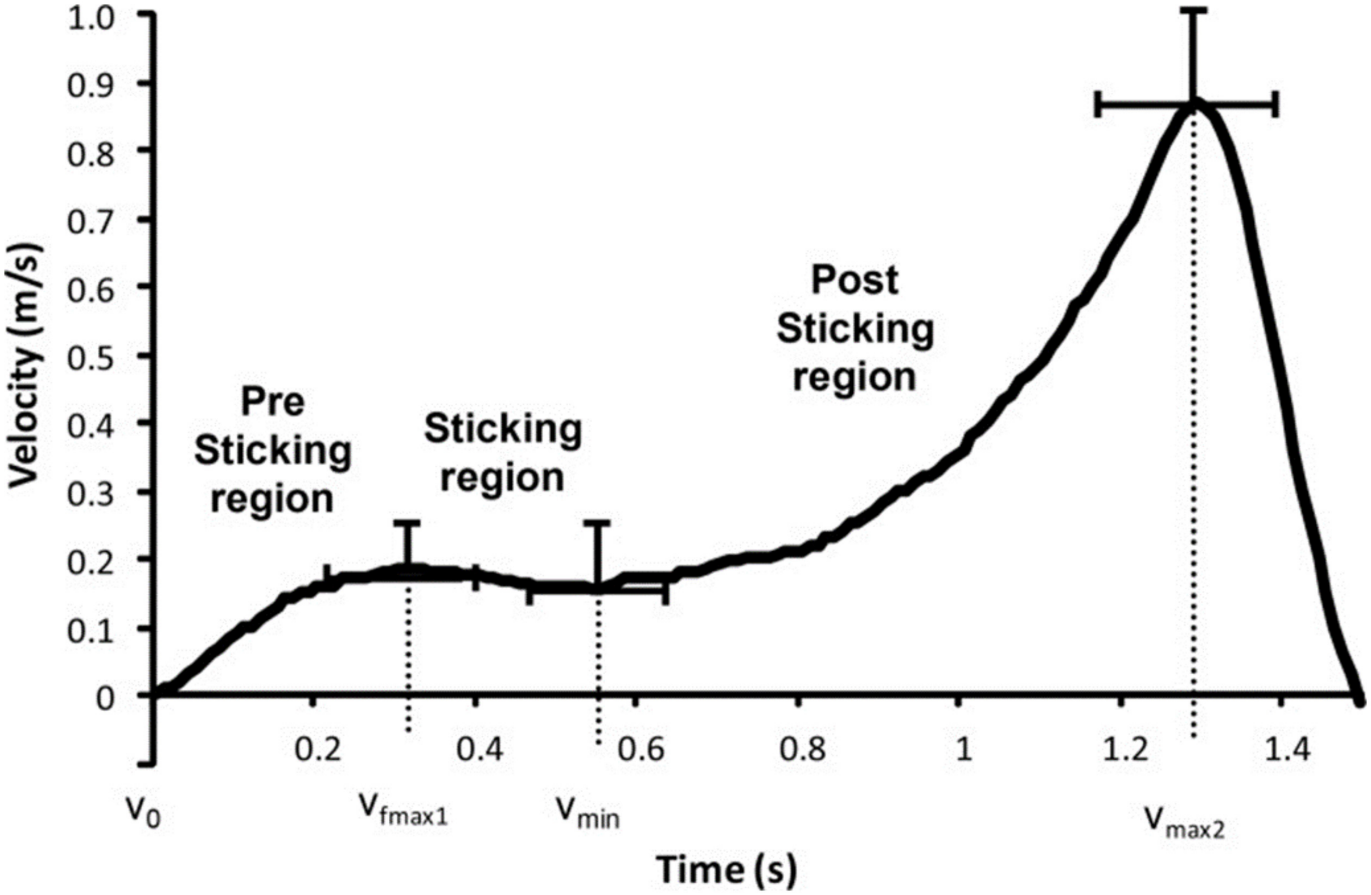
Mean ± SD vertical barbell velocity during the last repetition in 6-RM back squats with a pre-sticking, sticking, and post-sticking region and following events: lowest barbell height (v_0_), first peak barbell velocity (v_max1_), first located minimum barbell velocity (v_min_), and second peak barbell velocity (v_max2_). Adapted from van den Tillaar et al. (2014).

As observed in earlier studies at maximal and near maximal attempts, a sticking region [the region from maximal barbell velocity (v_max1_) to the first local minimum velocity of the barbell (v_min_)] occurs in the squat during the ascending phase (Figure 1) (Madsen and Mclaughlin, 1984; Elliott et al., 1989; van den Tillaar and Ettema, 2010; van den Tillaar and Sæterbakken, 2012; van den Tillaar et al., 2012, 2020, 2021; van den Tillaar and Larsen, 2020; Larsen et al., 2021).

van den Tillaar and Ettema (2009) found that in this region, failure during maximal lifts occurred. During the sticking region, it was hypothesized that the involved muscles in the bench press may be at a disadvantageous length to generate optimal force, forming a weak biomechanical region (Madsen and Mclaughlin, 1984; Elliott et al., 1989). However, for maximal back squats, it was found that the gluteus muscles increased activity, while the quadriceps muscles decreased activity in the sticking region (van den Tillaar, 2015). Another finding was that the timing of the minimal and peak angular velocities of the plantar flexion knee extension and hip extension movements were associated with the different events of the sticking region, indicating that the coordination between hip, knee, and ankle joint movements was associated with the events around the sticking region (van den Tillaar, 2015). This less optimal activity during the sticking region was confirmed by van den Tillaar et al. (2021) when comparing back squats at one-repetition maximum (1-RM) with isometric squats performed at 10 different heights of the ascending phase. They found that force output was lowest in the sticking region, which occurred between 0 and 15 cm from the lowest vertical barbell point (v_0_) in the isometric trials, supporting that sticking region is a poor biomechanical region. In addition, they found that at these different heights during the isometric trials the quadriceps myoelectric activity was maximal during the first 25 cm from the lowest barbell height and decreased with increasing height, whereas gluteus maximus increased myoelectric activity first at around 25 cm barbell height from v_0_. Therefore, the authors suggested that the combination of quadriceps activity at the lower barbell heights, co-contraction between the hip and knee extensors, together with an ineffective gluteus maximus position for force production, created a poor biomechanical region for force output. However, no analysis of joint kinetics was performed that could verify this statement.

Furthermore, Maddox and Bennett (2021) investigated net joint moments during the sticking region in back squats and how loading affected the barbell velocity and acceleration. The authors argued that there were three important factors for overcoming the sticking region in back squats. First, vertical barbell acceleration was a more prejudiced measure compared with barbell velocity. Second, squatting with submaximal loadings could cause similar knee and ankle net joint moments and contributions as squatting at maximal loads, but not for the hip joint, where load was the variable that increased hip net joint moments. Therefore, the authors suggested that since the net joint moments increased for the hip at maximal or supramaximal back squats, athletes should train their hip extensors to overcome these large hip net joint moments. Nevertheless, Maddox and Bennett (2021) only investigated how the joint moments affected moment contributions in the pre-sticking and sticking region. To the best of our knowledge, no studies have performed a full barbell, joint kinematics, joint kinetics (force, moments, moment arms) analysis in tandem with myoelectric activity at both the pre-sticking, sticking, and post-sticking regions. Also, to our knowledge, no studies have investigated and reported the kinematic and kinetic variables at maximal barbell deacceleration (d_max1_) during the ascending phase, which Maddox and Bennett (2021) pointed out could be of main importance for better understanding the sticking region. Investigating the joint kinematics, kinetics, and myoelectric activity for the hip, knee, and ankle joints could provide comprehensive knowledge and understanding about variables affecting the sticking region, and may be of value for athletes who strive to overcome the sticking region due to limitations in certain prime movers.

Therefore, the purpose of this study was to investigate barbell, joint kinematics, joint kinetics of hip, knee, and ankle in tandem with myoelectric activity around the sticking region in back squats. It was hypothesized that hip net joint moment, moment arm, and moment contribution would peak at d_max1_ and v_min_ despite lower ground reaction forces at d_max1_ due to peak deacceleration, indicating that the increased hip demands at these events may be a substantial contributor to the occurrence of the sticking region in back squats, as suggested by Maddox and Bennett (2021).

## Methods

### Experimental Design

To investigate the kinematics, kinetics, and myoelectric activity around the sticking region, the barbell kinematics of the ascending phase was analyzed in five events and three regions: The first event is when the lift changes from the descending to the ascending phase, which is from the lowest vertical height of the barbell, where the velocity is zero (v_0_). The second point is the first maximal barbell velocity (v_max1_), and the region between these points is called the pre-sticking region. The following region is described as the sticking region, and this is from v_max1_ to the first local minimal barbell velocity (v_min_). In this region also, the maximal barbell deceleration event (d_max1_) occurs. After the sticking region, the velocity increases again to the second maximal peak velocity (v_max2_), and the last region between v_min_ and v_max2_ is called the post-sticking region. The dependent variables, first and second peak, together with minimum and maximal hip, knee, and ankle angular velocities and their timings were collected as instantaneous variables with the same methods as van den Tillaar (2015). Moreover, barbell velocity, displacement, joint angles, both vertical and horizontal forces exerted to the ground, hip, knee, and ankle, net joint moments, moment arms, and moment contribution to total moment were collected at the events v_0_, v_max1_, d_max1_, v_min_, and v_max2_, whereas myoelectric activity was collected as means during the pre-sticking, sticking, and post-sticking regions during the last repetition of the 3-RM lifts.

### Participants

In this study there were 25 participants, 13 of whom were females (age: 24.2 ± 3.8years, height: 166 ± 4.8 cm, weight: 66.1 ± 8.9 kg, fat percentage: 24.7 ± 4.5%) and 12 of whom were males (age: 25.2 ± 2.9 years, height: 179.1 ± 5.9 cm, weight: 82.7 ± 7.2 kg, fat percentage: 17.2 ± 2.8%). To take part in the results, there were some inclusion criteria: (1) The females needed to squat 1.0 own bodyweight, and males needed to be able to lift 1.5 times their body weight in 1-RM with their favored back squat technique. (2) No illness or injury should be present that can lower maximum performance. (3) Participants had to achieve the depth necessity determined by the International Powerlifting International Powerlifting Federation (2020), which was that the top surface at the hip joint was below the knees in the lowest position. (4) All three familiarization sessions and the test session had to be conducted. (5) Age should be between 18 and 50 years. Additionally, during the 48 h before testing, the participants could not consume any alcohol or execute any resistance training on the legs. The participants were informed verbally and in writing of potential risk of participating in this study. Before the first familiarization session, a written consent of each participant was attained. The study fulfilled current ethical regulations for research and was accepted by the National Center for Research Data, in accordance with the latest revision of the Declaration of Helsinki.

### Procedures

To examine the kinematics, kinetics, and myoelectric activity around the sticking region, during a high-bar squat, a 3-RM test was used since it is a typical load used among powerlifters for increasing maximal strength (Baechle and Earle, 2008). Before the test session, three familiarization sessions were performed. This was to make sure of appropriate execution on the test day and to find the real 3-RM. In the first familiarization test, distance from the right acromion to the left acromion was measured to determine the stance width for each participant. Of the acromion length, 0.7 times was utilized as stance width and standardized throughout all familiarization sessions and on the testing day with a tape on both force plates. During this study, the external rotation on the foot was self-selected by the participants but standardized and monitored throughout. The depth through the end of the eccentric phase was measured and standardized with the depth requirement that the International Powerlifting International Powerlifting Federation (2020) has for an accepted back squat, and was measured in an axial direction from the ground and standardized through all familiarizations and the test session and marked with a horizontal band. The barbell placement was measured as the distance in an axial direction from the C7 spinous process of the vertebra to the barbell (distance: 0.4 ± 0.3 cm,), and the grip width was measured as the horizontal distance from the radius of the barbell to the first metacarpal (distance: 30 ± 5.3 cm). Both the stance width and barbell placement were identical throughout all familiarization and test sessions. Thereafter, every participant squatted three repetitions with 60% of predicted 3-RM. To find the participants' individual 3-RM, they were tested in their 1-RM in one of the familiarization sessions in a randomized order. Mean concentric barbell velocity was measured with a linear encoder (ET-Enc-02, Ergotest Technology AS, Langesund, Norway) to ensure that it was a true 3-RM from the load–velocity relationship. The calculation of mean ascending barbell velocity was done on the final repetition. Both the familiarization sessions and the test day began with a general warm-up, which involved three sets of six to 10 repetitions with an Olympic barbell (Rogue, Ohio power bar). During all three familiarization sessions, participants had 180 s of rest between warm-ups and 240 s between maximal lifting sets. The participants had at least 96 h of rest between the familiarization sessions and 120 h of rest between the test sessions. This was to avoid unwarranted fatigue that could influence the performance.

### Measurements

To measure the lifting time of the barbell, and the vertical displacement, a linear encoder (ET-Enc-02, Ergotest Technology AS, Langesund, Norway) was used and measured from the lowest point of the barbell (v_0_) with a resolution of 0.019 mm and 200 Hz sampling rate. To calculate the velocity of the barbell, the five-point differential filter with software (Musclelab version: 10.200.90.5095, Ergotest Innovation, Porsgrund, Norway) was utilized. Joint kinematics and kinetics, barbell displacement, and velocity were recognized at the five different events (v_0_, v_max1_, d_max1_, v_min_, and v_max2_) during the ascending phase of the squat at the last repetition. The linear encoder was synchronized with the EMG recordings utilizing a Musclelab 6000 system and analyzed by Musclelab v10.200.90.5095 software (Ergotest Technology AS, Langesund, Norway). Musclelab was also utilized to record myoelectric activity on the participants' dominant side in the different muscles: erector spinae iliocostalis, erector spinae longissimus, gluteus maximus, adductor longus, rectus femoris, vastus lateralis, vastus medialis, gluteus medius, semitendinosus, biceps femoris, gastrocnemius medialis, and soleus medialis. The different muscles' location and orientation were done according to SENIAM recommendations (Hermens et al., 2000) for proper muscle recordings. To lower skin impendence before self-adhesive electrodes (Dri-Stick Silver circular sEMG Electrodes AE-131, NeuroDyne Medical, USA) with 11 mm contact diameter, 20 mm center-to-center distance was placed on the right side on the 12 muscles with a sampling rate of 1,000 Hz, the participants' skin was shaved, rubbed with alcohol, and dried with paper. To lower noise, conductive gel (Signa Gel, Parker Laboratories Inc., Fairfield, NJ, USA) was put onto the electrodes. A preamplifier was used on the raw EMG signals for amplifying and filtering at high-pass and low-pass (500, 20 Hz) level. The common-mode rejection ratio was 106 dB. The mean RMS was calculated for the pre-sticking, sticking, and post-sticking regions. The participants executed a 5-s maximal voluntary isometric contraction (MVIC) squat at the same barbell placement, depth, and stance width as the lowest position achieved with the high-bar narrow stance for normalization. The barbell was attached to a squat rack, which can be corrected axially. The participants were directed to attain maximum force as rapidly as possible and sustain the force all the way through the trial. Mean RMS between 2 and 4 s was used to calculate the maximal EMG activity for normalization of the RMS signals of the different regions during the 3-RM lift.

To track reflective markers for motion capture data such as joint angles and angular velocities, a three-dimensional motion capture system (Qualisys, Gothenburg, Sweden) with eight cameras at a sampling rate of 500 Hz was utilized. Markers were placed on both sides of the body, except for the upper and lower hand, where the markers were placed on the dominant side. For the lower and upper and arm segment, markers were positioned on the radial and ulna styloid process and the lateral and medial epicondyle of the humerus. For the pelvis, markers were positioned on the posterior superior iliac spine and anterior superior iliac spine, creating a hip joint center and coda pelvis (Bell et al., 1987, 1990). For the thorax, markers were placed on the C7 spinous process of the vertebra, acromion, thoracal process 1 of the vertebra, the midpoint between the inferior angles of the most caudal points of the two scapulae, and sternum xiphisternal and sternum jugular notch joint (C-Motion, 2017). Markers for the foot and shank were positioned on the femoral lateral and medial epicondyle, first and fifth proximal phalanx, and the lateral and medial malleolus. Also, four markers were positioned on the barbell with a 20-cm distance. To track the three-dimensional ground reaction forces and enable inverse dynamics calculation, mediolateral to vertical force ratio and ground reaction force moment arms, two force plates (AMTI Multi-axis Force Transducer BP6001200-2000, Lexington, KY, USA; Kistler force plate, type 9260AA6, Winterthur, Switzerland) were integrated into the Qualisys motion capture system. The ground reaction force moment arms were calculated as the anterior–posterior distance between the joint centers and center of pressure.

Motion capture data were exported to C3D files for segment modeling and analyses in Visual 3D v6 software (C-motion, Germantown, USA). All computations from the model-based data were smoothed with a low-pass Butterworth filter at a cutoff frequency of 10 Hz. Joint angles for the torso, hip, knee, and ankle in the events v_0_, v_max1_, d_max1_ v_min_, and v_max2_ were calculated in distal to proximal orientation with a Cardan sequence in the order x–y–z. The x-, y-, and z-axes were created to mediolateral, anterior–posterior, and vertical directions, and the origin of the axes was set to the corner of the left force platform.

Joint angles for the hip knee and ankle were calculated as the angle between the distal and proximal segments, and torso angle was calculated as the angle between the torso segment and the lab. The three-dimensional joint moments for the hip, knee, and ankle were calculated, using inverse dynamics calculations in a resolute coordinate system. The joint moments calculated in this study are internal net joint moments, expressed as means and standard deviations at the events v_0_, v_max1_, d_max1_, v_min_, and v_max2_ with respect to the distal segments' resolute coordinate system. The reported net joint moments data were summed between the right and left segments. Net joint moments from the sagittal plane are flexion and extension moments and net joint moments from the frontal plane are abduction and adduction moments. Net joint moments from the analyzed planes were normalized to the participants' mass using default normalization and expressed as Nm/kg. When calculating the hip, knee, and ankle contributions to the total net joint moments, all abduction and adduction values were normalized into positive values. To calculate and track the mediolateral to vertical force ratio as percentage at each event, ground reaction force data x-values were divided by the ground reaction force data z-values and multiplied by 100. Mediolateral to vertical force ratio was calculated to enhance understanding of the development of adduction and abduction net joint moments during the events.

### Statistics

Normality was tested using the Shapiro–Wilks test. To assess the difference in kinetics between the events v_0_, d_max1_ v_max1_, v_min_, and v_max2_, together with the differences in myoelectric activity between the pre-sticking, sticking, and post-sticking regions, a one-way repeated measures analysis of variance (ANOVA) was performed. For differences between sexes in barbell kinematics, a repeated 2 (sex: men, women) × 2 (event: v_0_, d_max1_, v_max1_, v_min_, and v_max2_) ANOVA was performed. Holm–Bonferroni *post-hoc* tests were used to identify where potential differences in kinetics and myoelectric activity occurred. If assumption of the sphericity was violated, the Greenhouse–Geisser adjustments of *p*-values were reported. All results are presented as mean ± standard deviations. Effect sizes were evaluated with $\eta_{\text{p}}^{2}$ (partial eta squared), where <0.01–0.06 constitutes a small effect, <0.06–0.14 a medium effect, and >0.14 a large effect (Cohen, 1988). The alpha level of significance was set at *p* < 0.05. Statistics were analyzed in SPSS version 27.0 (IBM Corp., Armonk, NY, USA).

## Results

**Figure 2:**
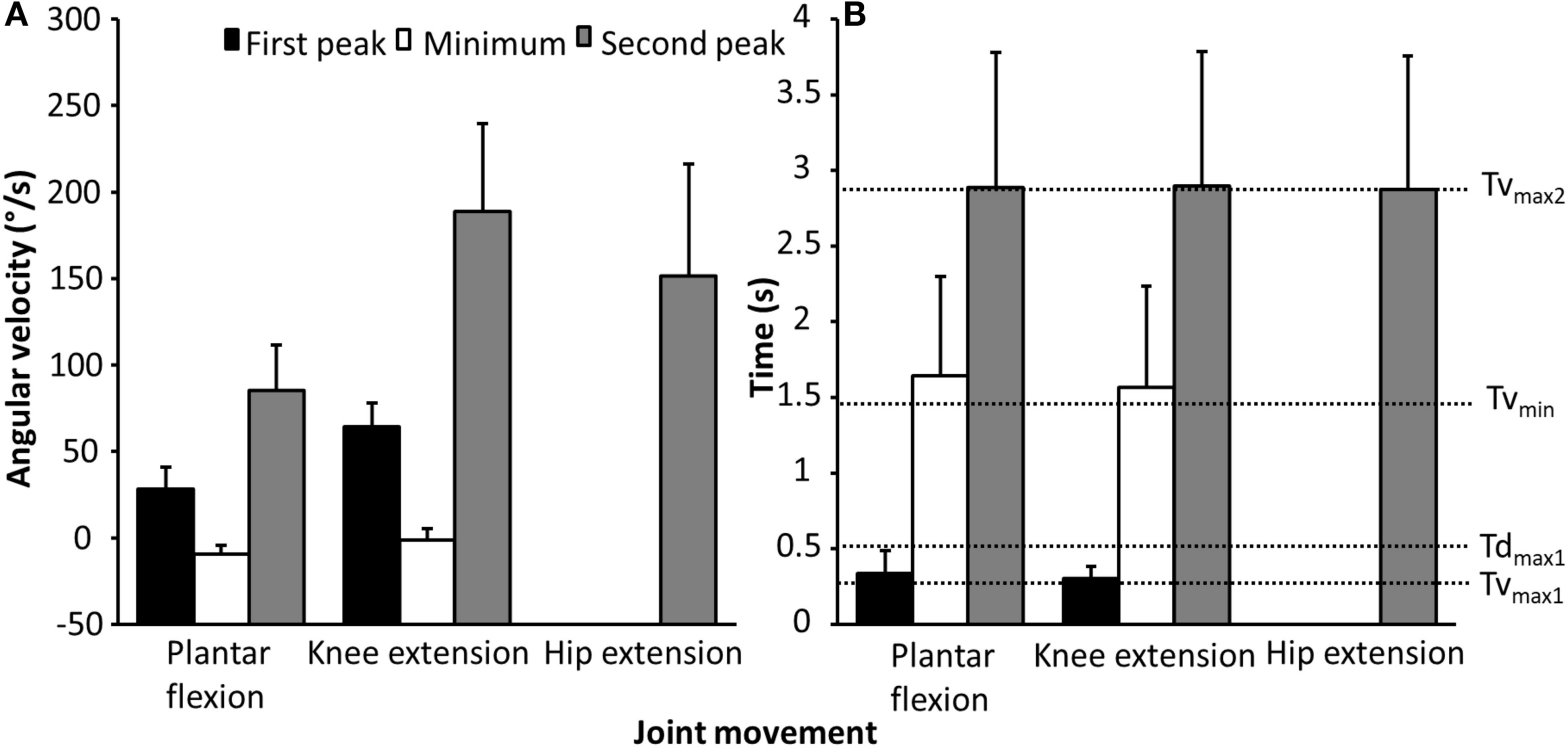
Mean ± SD **(A)** first and second peak and minimum angular velocity of the plantar flexion, knee, and hip extension, **(B)** timing of the first and second peak, and minimum angular velocity of the plantar flexion, knee, and hip extension during the last repetition in 3-RM back squats.

The participants lifted 90.3 ± 26.5 kg., whereas the men lifted 121 ± 9.3 kg and the women 72.7 ± 19.8 kg. No significant differences were found between the sexes for barbell kinematics in any of the events (*F* ≤ 2.7, *p* ≥ 0.14, η^2^ ≤ 0.11). Descriptive data of the joint and barbell kinematics are presented in Table 1 and Figure 2.

**Table 1:** Mean ± SD barbell displacement, time, and velocity in the events v_0_, v_max1_, d_max1_, v_min_, and v_max2_ together with torso flexion, hip flexion, hip abduction, and external rotation, knee flexion angle, plantar flexion angles during the last repetition in 3-RM back squats.

**Event**	**v_**0**_**	**v_**max1**_**	**d_**max1**_**	**v_**min**_**	**v_**max2**_**
Velocity (m/s)	0	0.34 ± 0.07	0.21 ± 0.06	0.11 ± 0.06	0.82 ± 0.21
Displacement (m)	0	0.06 ± 0.01	0.17 ± 0.04	0.24 ± 0.06	0.58 ± 0.06
Time (s)	0	0.24 ± 0.06	0.52 ± 0.2	1.48 ± 0.29	2.8 ± 0.92
**Angles (°)**					
Torso (°)	48.6 ± 11.5	51.9 ± 12.6	55.6 ± 13.1	56.4 ± 13.8	32.5 ± 8.8
Hip flexion	111.8 ± 7.8	108.3 ± 7.8	101.6 ± 12.9	92.5 ± 11.4	49.6 ± 10.2
Hip abduction	13.8 ± 7.6	10.3 ± 7.8	6.8 ± 7.8	5.7 ± 6.8	6.1 ± 4.4
Hip external rotation	7.0 ± 5.3	6.2 ± 5.5	4.7 ± 5.9	1.8 ± 5.6	−10.2 ± 6.4
Knee flexion	125.3 ± 6.6	115.2 ± 6.9	99.2 ± 11.5	84.7 ± 9.6	49.2 ± 5.6
Plantar flexion	105.2 ± 5.4	101.9 ± 4.6	97.1 ± 6.2	93.2 ± 6.0	87.1 ± 4.8

**Figure 3:**
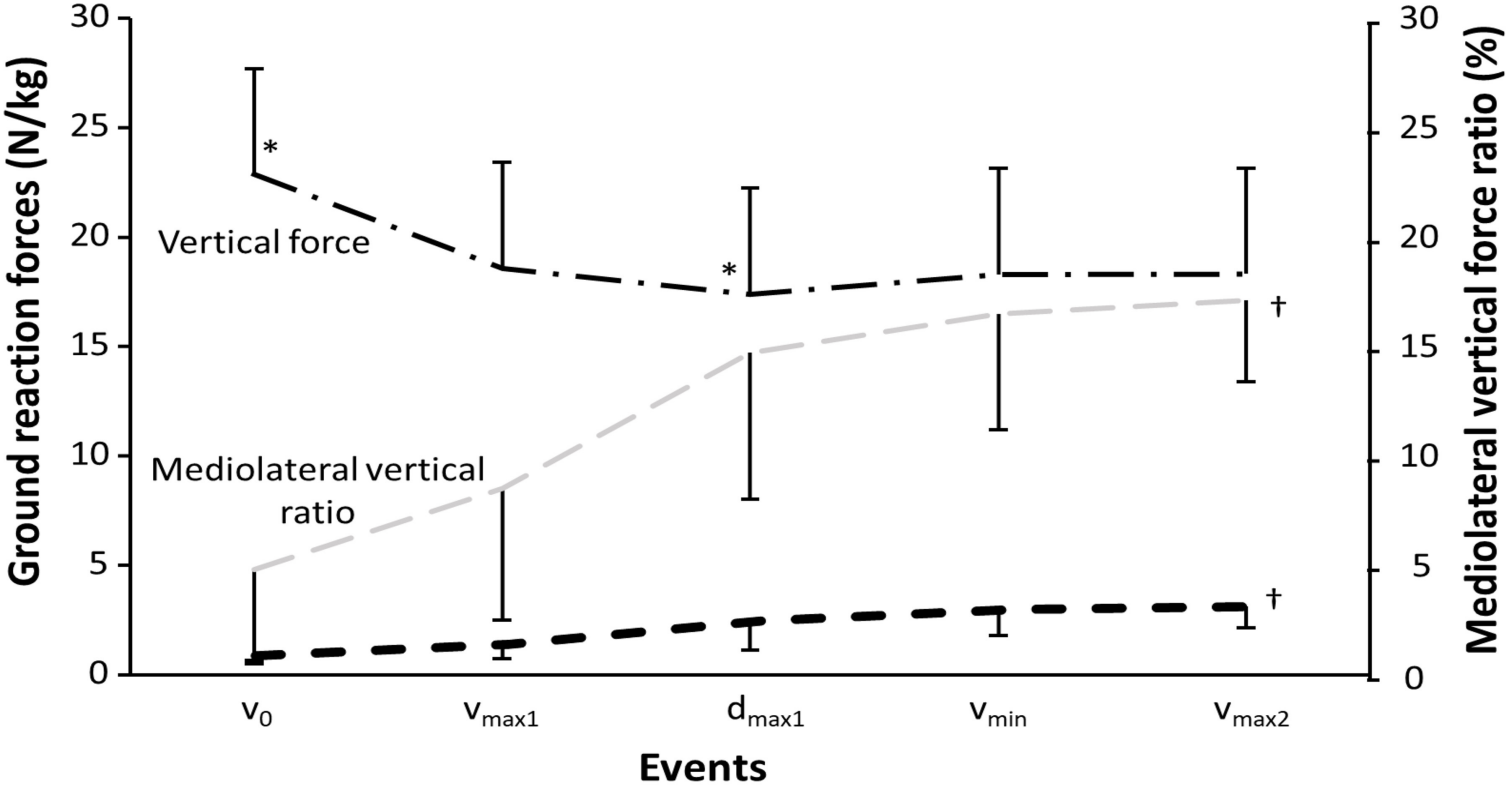
Mean ± SD vertical and horizontal ground reaction forces together with mediolateral to vertical force ratio. * indicates a significant difference with all other events on a *p* ≤ 0.05 level. † indicates a significant difference between all events for this variable on a *p* ≤ 0.05 level.

A significant effect was found for event upon vertical and horizontal ground reaction force together with mediolateral to vertical force ratio (*F* ≥ 26.7, *p* ≤ 0.001, η^2^ ≥ 0.57) (Figure 3). *Post-hoc* tests showed that vertical ground reaction forces decreased from v_0_ to all other events, while mediolateral forces increased during each event, resulting in increased mediolateral to vertical force ratio during each event. Also, vertical ground reaction forces were lower at d_max1_ compared with v_max1_ and v_min._ This resulted in knee abduction and hip adduction moment in v_0_ and v_max1_, which changed to knee adduction and hip abduction moment in d_max1_, v_min_, and v_max2_, together with ankle abduction moments during all events. Anteroposterior forces were 0.4 ± 0.3, 0.4 ± 0.4, 0.3 ± 0.2, 0.3 ± 0.2, and 0.4 ± 0.4 N/kg at the events v_0_, v_max1_, d_max1_, v_min_, and v_max2_, with no significant differences between the events (*F* = 2.7, *p* = 0.15, η^2^ = 0.11).

**Figure 4:**
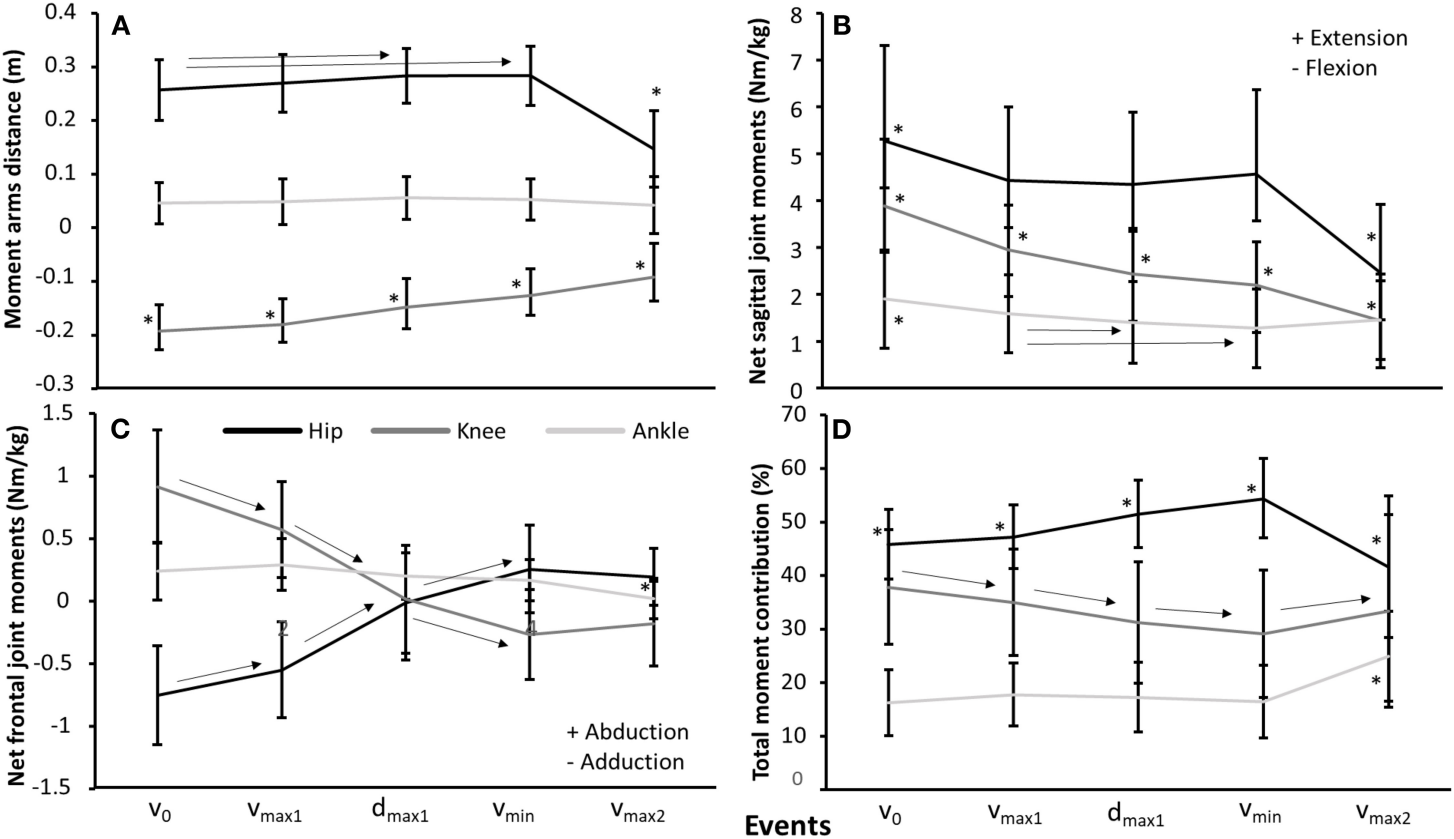
Mean ± SD **(A)** moment arms, **(B)** sagittal net joint moments, **(C)** frontal net joint moments, and **(D)** net moment contribution to total net moment for the hip, knee, and ankle joints in the events v_0_, v_max1_, d_max1_, v_min_, and v_max2_ during the last repetition in 3-RM back squats. * indicates a significant difference from all other events on a *p* ≤ 0.05 level. → indicates a significant difference between these two events on a *p* ≤ 0.05 level.

Furthermore, a significant effect was found for event upon hip and knee moment arms (*F* ≥ 42.2, *p* ≤ 0.001, η^2^ ≥ 0.68), but not ankle moment arm (*F* = 3.1, *p* = 0.09, η^2^ = 0.13) (Figure 4A). *Post-hoc* tests revealed that hip moment arms increased from v_0_ to d_max1_ and v_min_ before decreasing in v_max2_, while knee moment arms decreased from event to event. For hip, knee, and ankle sagittal and frontal net moments together with moment contribution to total net moment, a significant effect for event was found (*F* ≥ 10.1, *p* ≤ 0.001, η^2^ ≥ 0.34) (Figures 4B–D). *Post-hoc* tests showed that hip extension moment decreased from v_0_ to v_max1_, where the hip extension moment was stable through the sticking region before decreasing again in v_max2_. Furthermore, knee extension moment decreased through each event, while ankle plantar flexion moment decreased from v_0_ to all other events, and from v_max1_ to d_max1_ and v_min_ (Figure 4B). The frontal moments, hip adduction, and knee abduction moments were observed in v_0_ and v_max1_, which changed to hip abduction and knee adduction moments in d_max1_ before increasing further in v_min_. Furthermore, an ankle abduction moment was observed during all events, which decreased at v_max2_ compared with the other events (Figure 4C). All these resulted in the hip contribution to the total moment increasing almost linearly at each event in the sticking region, with a peak at v_min_ before decreasing again in v_max2_, whereas knee contribution showed the opposite: decreased from v_0_ to v_max1_, d_max1_, and v_min_ before increasing in v_max2_. Ankle contribution remained stable at the four first events before increasing at v_max2_ (Figure 4D).

**Figure 5:**
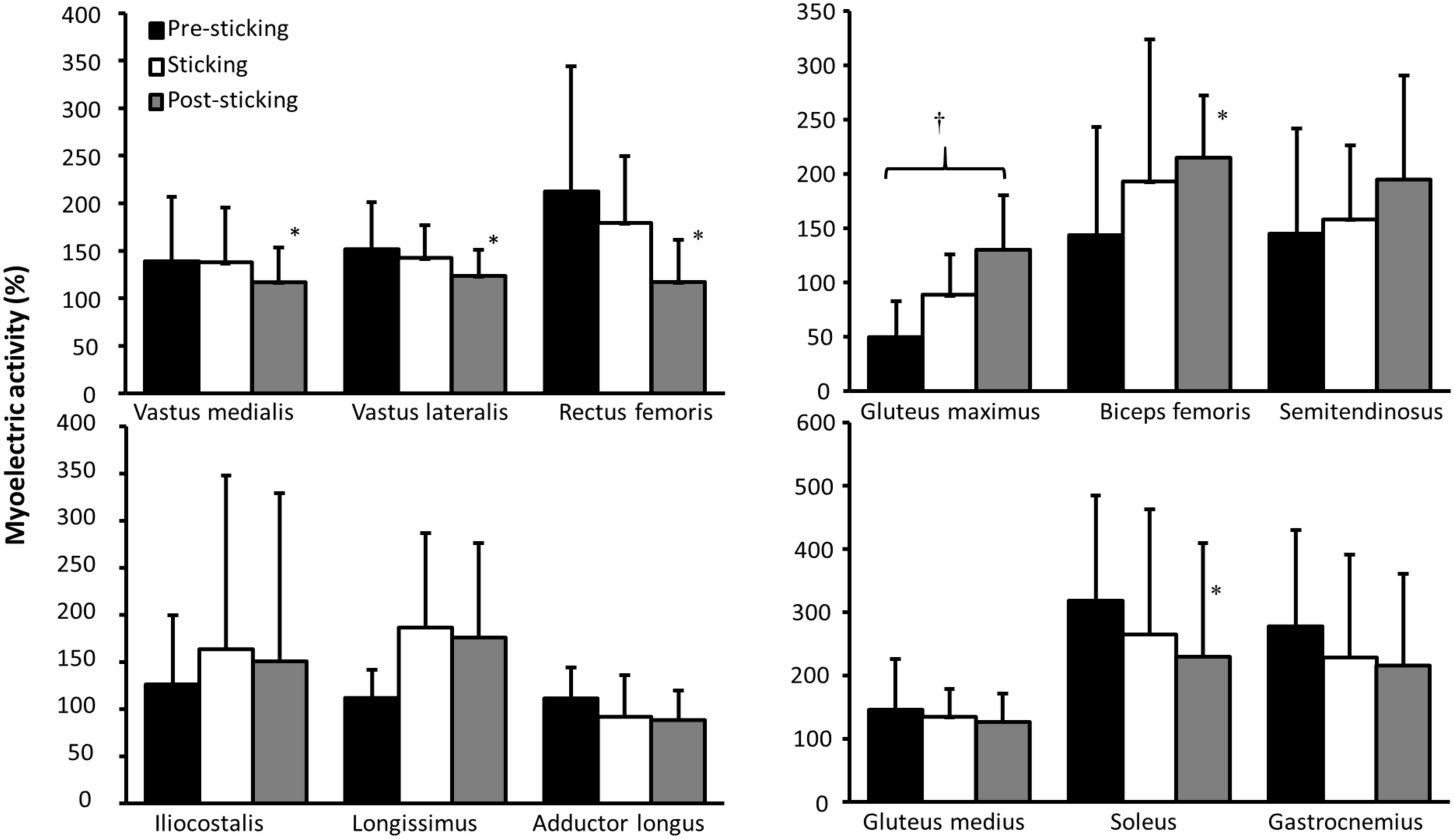
Mean ± SD normalized myoelectric activity for the erector spinae iliocostalis, erector spinae longissimus, adductor longus, gluteus maximus, biceps femoris, semitendinosus, vastus medialis, vastus lateralis, rectus femoris, gluteus medius, soleus, and gastrocnemius in the pre-sticking, sticking, and post-sticking regions during the last repetition in 3-RM back squats. † indicates a significant difference between these two regions on a *p* ≤ 0.05 level. * indicates a significant difference between this region and all other regions on a *p* ≤ 0.05 level.

A significant effect of region upon myoelectric activity was found for gluteus maximus, biceps femoris, vastus medialis, vastus lateralis, rectus femoris, and soleus (*F* ≥ 3.6, *p* ≤ 0.037, η^2^ ≥ 0.15), but not for the erector spinae iliocostalis, erector spinae longissimus, adductor longus, semitendinosus, gluteus medius, or gastrocnemius (*F* ≥ 3.2, *p* ≤ 0.066, η^2^ ≥ 0.13) (Figure 5). *Post-hoc* test revealed that gluteus maximus increased myoelectric activity from pre-sticking to post-sticking region, whereas the biceps femoris increased myoelectric activity from pre-sticking and sticking to post-sticking region. The opposite occurred for the quadriceps muscles together with the soleus muscle, where myoelectric activity decreased from pre-sticking and sticking to post-sticking region.

## Discussion

The purpose of this study was to investigate barbell, joint kinematics, joint kinetics of hip, knee, and ankle in tandem with myoelectric activity around the sticking region in back squats. The main findings were that hip and knee extension together with plantar flexion movements were concomitant with the timing of the events v_max1_, v_min_, and v_max2_, as previously reported by van den Tillaar (2015) (Figure 2). Furthermore, torso inclination peaked in d_max1_ and v_min_ (Table 1). Whereas hip moment arm and moment contribution increased, knee moment arm and knee contribution decreased in d_max1_ and v_min_ (Figure 4), which was in accordance with our hypothesis. Furthermore, quadriceps and soleus myoelectric activity remained stable in the pre-sticking and sticking regions before decreasing in the post-sticking region, whereas the gluteus maximus and biceps femoris increased myoelectric activity from the pre-sticking to the post-sticking region (Figure 5).

A clear sticking region was observed during the last repetition among all of the participants, which is in accordance with previous studies investigating the sticking region in back squats (Escamilla et al., 2001a; van den Tillaar, 2019; Maddox et al., 2020; van den Tillaar et al., 2020, 2021; Maddox and Bennett, 2021). d_max1_ occurred at ~50% barbell height between the events v_max1_ and v_min_, whereas the sticking region started after ~0.06 m and 0.24 s, and ended at 0.24 m and 1.48 s. van den Tillaar (2015) reported similar barbell kinematics for the sticking region (0.08 and 0.22 m), the time spent in the sticking region was much shorter (0.21 vs. 1.24 s) than in the present study, which may perhaps be explained by the different % of 1-RM used (3-RM vs. 6-RM) in the studies. When comparing other kinematic parameters between this study and van den Tillaar (2015), such as knee flexion, hip flexion, and plantar flexion angles, sticking region started at 10.1° vs. 13.6°, 3.5° vs. 5.5°, and 4.1° and 6.5°, and ended at 40.6° vs. 34.8°, 19.3° vs. 22.5°, and 12° and 12.8° from v_0_. These similar findings in joint angles of the start and end of sticking region for both studies indicate that sticking region could be angle specific because less force could be produced due to large external moments and moment arms in combination with an ineffective internal moment arm of the gluteus maximus, as speculated by van den Tillaar (2015).

Furthermore, whereas peak hip, knee, and ankle net sagittal joint moments were produced at v_0_, the sticking region started at 0.06 m barbell height after 0.24 s. This could be explained by potentiation of the prime movers, which is caused by the stretch-shortening contraction movement, as shown by van den Tillaar et al. (2021). This stretch-shortening contraction makes it possible to produce more force during the early shortening period. This effect has been reported previously to disappear after around 0.3 s in such resistance exercises (Walshe et al., 1998), which is around where the sticking region started in the present study.

Since the diminishing potentiation is responsible for the start of the sticking region, the sticking region is a region in which less vertical force (Figure 3) can be produced (van den Tillaar et al., 2021). This is mainly caused by the constant hip moment in this region. As observed, the sagittal knee and ankle moments decreased during the sticking region, resulting in an increased hip moment contribution (Figure 4D). The ascending squat phase starts with the ankle plantar flexion and knee extension movement, which peaks at v_max1_, while hip extension does not occur much during the first part of the ascending phase (Table 1; Figure 2). A result of these movements is the increase and decrease in, respectively, the hip and knee moment arms (Figure 4A). That the movements started with the ankle and knee movements is probably due to the moment of inertia of the different segments. The foot and lower limbs have a lower moment of inertia than the trunk with the added barbell load. When the barbell is lowered this large moment of inertia causes first movements in the lower limb, while the hip and trunk are delayed in the ascending phase. This is observed by the increased torso forward lean from v_0_ to v_max1_, d_max1_, and v_min_ with 3.3, 7, and 7.8°. The large moment of inertia of the torso increases the time to accelerate and thereby hip flexion angular velocity later in the ascending phase, thus, putting larger demands from the knee extensors in the pre-sticking region to the hip extensors in the sticking and post-sticking regions. The peak hip moment arms observed at d_max1_ and v_min_ coincided with peak hip contributions to total moment at the same events, which were 51.5 and 54.4% at d_max1_ and v_min_. Moreover, knee moment contributions peaked in v_0_ with 37.8% and were reduced to 31.1 and 29.1% in d_max1_ and v_min_. Furthermore, due to the large hip flexion and forward trunk lean at the first part of the ascending phase, the large gluteus muscle length gives a mechanical disadvantage to exert force during the pre-sticking and sticking regions (Robertson et al., 2008; van den Tillaar et al., 2021). This lower myoelectric activity of the gluteus maximus combined with the peak hip contributions observed in the sticking region could therefore be confirmed as an explanation on why the sticking region occurs in back squats. The gluteus maximus and biceps femoris increased myoelectric activity from sticking to post-sticking region in the present study, which was also observed in earlier studies of van den Tillaar (2019), van den Tillaar et al. (2021). van den Tillaar et al. (2021) found that at around 0.25-m barbell from v_0_ height, gluteus maximus and hamstring activity increased, which was concomitant with the start of the post-sticking region. This is a further indication that the gluteus maximus, in collaboration with the hamstring muscles, is responsible for ending the sticking region. At around this barbell height, the force–length relationship of these muscles is probably more effective so that they can increase their pulling forces. However, no modeling of these muscles that could confirm this statement is performed in the present study.

As expected, vertical ground reaction forces decreased in d_max1_ due to peak deacceleration. Interestingly at the same time, the mediolateral forces exerted against the ground increased during each event, and resulted in almost a doubling in mediolateral to vertical force ratio from v_max1_ to d_max1_ (8.5 vs. 14.7%). This created a shift in frontal net joint moments at d_max1_. Whereas hip adduction and knee abduction moments were observed in v_0_ and v_max1_, these shifted to hip abduction moments together with knee adduction moments in d_max1_ v_min_, and v_max2_. It is speculated that this shift in frontal moments for both the hip and knee joint could be a contributor to first peak deacceleration because of shifting some of the demands from the hip adductors to the hip abductors around this event. Nevertheless, our EMG data on the adductor longus and gluteus medius could not confirm this speculation. However, it may be that increasing the stance width could influence how these muscles contribute to hip adduction and abduction moments because of an increased mediolateral to vertical force ratio for the wide stance back squat, as reported by Lahti et al. (2019).

## Limitations

Inverse dynamics calculate net joint forces and not joint contact forces, which means that this method neglects muscle forces, which are often a primary source of joint loading (Vigotsky et al., 2019). Therefore, further research should use musculoskeletal modeling techniques to calculate joint contact forces and moments experienced by the musculoskeletal system. Also, this study only investigated kinematics and kinetics in the high-bar back squat, whereas both stance width and barbell placement have been shown to affect lifting performance (Escamilla et al., 2001a; Swinton et al., 2012; Glassbrook et al., 2017, 2019; Lahti et al., 2019; van den Tillaar et al., 2020). Therefore, further studies should also investigate how changing barbell placement together with stance width could change the kinematics, kinetics, and myoelectric activity around the sticking region in back squats. Furthermore, the participants in this study were recreationally trained lifters and not powerlifters. Therefore, our findings may not be generalizable to this cohort, and further studies should investigate how the kinetics develop during maximal back squats among powerlifters.

## Conclusions

Ground reaction force output decreased, and hip moment arm and hip contribution to total moment increased due to increased torso forward lean at d_max1_. Also, gluteus maximus and biceps femoris myoelectric activity peaked first in the post-sticking region. Therefore, our findings indicate that the large hip extension moment arms and moment contribution to total moment together with a lower gluteus maximus and hamstring myoelectric activity during the sticking region contribute to a poor biomechanical region, and thereby to the sticking region, among recreationally trained lifters during 3-RM back squats.

## Data Availability Statement

The raw data supporting the conclusions of this article will be made available by the authors, without undue reservation.

## Ethics Statement

Ethical review and approval was not required for the study on human participants in accordance with the local legislation and institutional requirements. The patients/participants provided their written informed consent to participate in this study.

## Author Contributions

SL, EK, and RV designed the experiment, performed the experiments, analyzed the data, prepared figures and/or tables, authored or reviewed drafts of the paper, and approved the final draft together. All authors contributed to the article and approved the submitted version.

## Conflict of Interest

The authors declare that the research was conducted in the absence of any commercial or financial relationships that could be construed as a potential conflict of interest.
